# Cross-sectional study on the adherence to malaria guidelines in lakeshore facilities of Buyende and Kaliro districts, Uganda

**DOI:** 10.1186/s12936-018-2577-x

**Published:** 2018-11-19

**Authors:** Henry Kaula, Sylvia Kiconco, Luigi Nuñez

**Affiliations:** 1 International Health Sciences University, Kampala, Uganda; 2 PACE Uganda, PSI, Kampala, Uganda

**Keywords:** Uganda, Malaria, Integrated Management of Malaria, Adherence, Kaliro, Buyende, Training

## Abstract

**Background:**

Uganda adopted the Integrated Management of Malaria (IMM) guidelines, which require testing all suspected cases of malaria prior to treatment and which have been implemented throughout the country. However, adherence to IMM guidelines has not been explicitly investigated, especially in lakeshore areas such as Buyende and Kaliro, two districts that remain highly burdened by malaria. This study assesses the level of adherence to IMM guidelines and pinpoints factors that influence IMM adherence by health providers in Buyende and Kaliro. A cross-sectional study among 197 patients and 26 healthcare providers was conducted. The algorithm for adherence to IMM guidelines was constructed to include physical examination, medical history, laboratory diagnosis, and anti-malarial drug prescription. Adherence was measured as a binary variable, and binary regression was used to identify factors associated with adherence to IMM guidelines.

**Results:**

Only 16 (8.1%) of the 197 patients had their medical history and physical examinations taken, while the majority (65.5%) of the patients were recommended for malaria (laboratory) testing. Regarding adherence to prescription guidelines, 127 (64.5%) of the patients received artemisinin combination therapy (ACT) drug prescription. On the other hand, 18.6% of those who tested negative received an ACT drug/prescription and 10.1% tested positive but did not receive an ACT drug or prescription. Overall adherence to IMM guidelines was only 3.1%. The only factor that significantly influenced adherence to IMM guidelines was training; healthcare providers who had attended recent training on these guidelines were almost three times more likely to adhere to the IMM guidelines compared to those who had not attended recent training (OR = 2.858, 95% CI 1.754–4.659).

**Conclusions:**

The findings indicate very low levels of adherence to IMM guidelines among healthcare workers in the lakeshore areas of Kaliro and Buyende districts. Since adherence was independently influenced, majorly by training healthcare workers on these guidelines, recommendations include facilitating training on IMM guidelines throughout Uganda.

## Background

Although sub-Saharan Africa continues to bear the largest burden of malaria with 88% of all cases and 9 in every 10 deaths globally, recent studies show that malaria is on the decline in the African region; there was a decline of 48% in the number of cases and 66% in deaths between 2000 and 2015 [1]. These declines may be attributed to improved access to malaria prevention and control tools, such as insecticide-treated bed nets (ITNs), indoor residual spraying (IRS), and artemisinin-based combination therapy (ACT). To consolidate the achievements in malaria prevention and control, the World Health Organization (WHO) updated its framework concerning malaria by requiring all countries where malaria is endemic to ensure that all suspected cases of the disease are tested via microscopy or rapid diagnostic test (RDT) and that every confirmed case is treated with a quality-assured anti-malarial drug [2].

Following the update of the WHO Framework for malaria prevention and control in 2012, Uganda, similarly to other countries where malaria is endemic, adopted these updated policies [3]. Uganda updated its national malaria treatment guidelines that same year to indicate first-line treatment and their alternatives for all cases of malaria, which includes any artemisinin-based combinations that has been recommended by the WHO and Ministry of Health (MOH) and is registered with the National Drug Authority. Adherence to the treatment guidelines has numerous benefits including improved use of anti-malarial drugs and reductions in the current high costs of ACT. It is, therefore, important that healthcare providers in both public and private sectors adhere to these guidelines for effective control of the disease.

Despite these efforts, Uganda has the third highest number of deaths from malaria and some of the highest recorded rates of malaria transmission in Africa, especially in areas around Lake Kyoga [4]. Both Buyende and Kaliro, two districts bordering Lake Kyoga, have hyperendemic transmission intensity rates (≥ 75%) for malaria at 85 and 100% of their populations, respectively. Meanwhile, these two districts are among those districts specifically targeted with heavy malaria interventions for being hyperendemic areas [5]. Despite many interventions, such as distributing free ITNs and increasing access to malaria diagnostic services and ACT by the Government of Uganda in the two districts, the area remains highly malaria burdened [5].

Several factors, including adherence to Integrated Management of Malaria (IMM) guidelines, may be majorly contributing to this consistent burden. Poor adherence, for example, can lead to increased malaria burden, parasite resistance, resource wastage, and treatment failure. This research sought to determine the level of adherence and associated factors to IMM guidelines among healthcare providers in Buyende and Kaliro. Findings may assist development partners and policy makers in identifying gaps related to IMM adherence and avert possible consequences, such as drug resistance, loss of lives and increased medical costs.

## Methods

### Study area

This study was conducted in the lakeshores of the Buyende and Kaliro districts in Uganda; lakeshores were defined as parishes that share borders with Lake Kyoga in Buyende and Kaliro districts [6]. Both Buyende and Kaliro are in Busoga, a sub-region in Eastern Uganda and surrounded by Lake Kyoga. Buyende, difficult to reach due to poor transport infrastructure, has a total population of 323,100 [7]. Kaliro has a total population of 236,200 [7]. Global positioning system (GPS) coordinates of the study facilities were taken, and Global Information System (GIS) software was used to generate a map of these areas.

### Study design

This study followed a cross-sectional design where data were collected from selected health facilities in September and October 2016 using a structured questionnaire. Primary data including socio-demographics, illness diagnoses, and medicine prescriptions were collected from patients and/or their attendants exiting the selected health facilities. Where available, the researcher or research assistant looked at patients’ medical forms to triangulate the information collected through the interview. Additionally, data on attitudes was collected from healthcare providers who were directly involved in malaria management in the selected health facilities.

### Study exposure and outcome

Exposure variables in this study were healthcare provider factors, such as cadre of healthcare providers, age, gender, level of education, training on national malaria guidelines, and health facility-related factors, including the type of facility and ownership. These factors were examined to see if they influence adherence to IMM guidelines. The individual health worker was linked to all the patients he/she had treated in order to establish whether the health worker had adhered to the guidelines. The study outcome was level of adherence to IMM guidelines. Adherence was defined as the proportion of diagnosis and prescriptions made in line with the national malaria management guidelines.

### Sample size and participant selection

Using the Leslie Kish 1965 formula [8],

$$ {\text{n }} = \, \left( {{\text{Z}}_{0. 9 5} } \right)^{ 2} \left( {{\text{P}}\left( { 1- {\text{P}}} \right)/{\text{D}}^{ 2} } \right) $$

with 95% confidence interval (i.e. Z_0.95_ = 1.96), 31% proportion (P) for fever in Uganda [9], and absolute precision (D) of 5%, a total sample of 329 participants was considered sufficient for this study. Buyende district has a total of 23 health facilities (5 at the lake shores) and while Kaliro has 21 (6 at the lakeshores) [6]. This study included all health facilities (11) along the shores of Lake Kyoga, 5 of which were in Buyende and 6 in Kaliro. These were the only ones that had information regarding the number of patients with fevers treated at each facility prior. Exit interviews were used, and every patient participant who met the inclusion criteria was included. Health workers involved in the diagnosis and treatment of suspected malaria were also included in the study. Private health facilities were anticipated to have a client flow of 10–20 clients a day while public health facilities had at least 50 clients. A total of 286 patients were screened; 223 were eligible, i.e., seeking treatment for illness including fever. Of the 223 eligible caregivers, 11% (26) declined to participate in the study. Extending the enrollment for this study was not possible due to insufficient funding.

### Data collection

Data were collected electronically using the Open Data Kit (ODK) software installed on Samsung Galaxy smart phones. Each phone was enabled to capture GPS coordinates to provide facility locations. The client/patient caregiver questionnaires were programmed and uploaded using ODK. The programmed tool had built-in validation steps to ensure no errors were made. Adult patients and caregivers of children below the age of 15 years were interviewed on exit from selected facilities after seeking diagnosis and treatment of fever. The data collectors were positioned at the exit of the outpatient side of the health facility to interview the patient participants while following interview protocol procedures to obtain the required information. Information regarding laboratory diagnosis and prescription was verified from a patient’s medical form or book while clinical diagnosis information was as reported by the participant. Similarly, questionnaires were administered to health workers who directly managed patients with suspected malaria.

### Data management and analysis

The data collected were downloaded as a Microsoft Excel workbook and then exported to SPSS version 17 for analysis. For univariate analysis, each individual variable was summarized using frequency tables, charts and graphs. For bivariate analysis, each independent variable was cross-tabulated with the dependent variable (adherence to national malaria management guidelines) to establish possible associations. The Chi Square test, or Fisher Exact Test (FET) for cases when the expected number was less than 5, was used to determine association between these categorical variables at a statistical significance level of 0.05. For multivariate level analysis, binary regression was conducted on those highly significant independent variables to determine independent factors’ influence on adherence to the national malaria guidelines, with 95% confidence intervals for the odds ratios and significance levels of 0.05. Data quality was ensured through comprehensive training of research assistants, pre-testing of tools, and constant supervision.

## Results

### Baseline characteristics of the study population

From Table 1, a total of 286 patients were screened at the facility exit after receiving treatment. Nearly half (47.9%) of the patients screened were from Buyende, and the rest (52.1%) were from Kaliro; 67.5% of the screened patients were female; 78.0% sought treatment for an illness that included fever, and 57.0% of this sub-set sought treatment for themselves. Nearly all (96.4%) of the screened patients were seeking treatment for a current episode of fever for the first time at the surveyed health facilities. The total number of patients or caregivers who consented to participate in the study was 197 (88.3%). Every patient who consented was seeking treatment for an illness that included fever, and only this sub-group was considered for further analysis, as is presented in the Tables. Patients’ average age was 19.6 (± 18.1) years.

**Table 1:** Baseline information of the study participants (n = 286)

Variable	Frequency	Per cent
Number of patients per district		
Buyende	137	47.9
Kaliro	149	52.1
Total	286	100.0
Distribution of gender		
Female	193	67.5
Male	93	32.5
Total	286	100.0
Sought treatment for an illness that included fever		
No	50	17.5
Yes	223	78.0
Did not respond to the question	13	4.5
Total	286	100.0
For whom care/treatment was sought		
Self	127	57.0
Child	94	42.1
Both self and child	2	0.9
Total	223	100.0
Distribution of age		
5 years old and below	82	41.6
Above 5 years old	115	58.4
Total	197	100.0

Source: primary data

### Level of adherence to malaria diagnosis guidelines

Table 2 shows level of adherence to malaria diagnosis guidelines, as reported by patients. Most health providers enquired about the patient’s age (97.0%), symptoms of the illness and when it began (94.9%), and history of fever (80.2%). However, only 95 (48.2%) of the patients were reportedly asked about signs of severe illness, including headaches, convulsions, and vomiting. Regarding physical examinations, only 28.9 and 22.3% of the 197 patients had their body weight and temperature taken using a weighing scale and a thermometer, respectively. The number of patients who reported that their health providers conducted both medical history and physical examinations was only 16 (8.1%).

**Table 2:** Level of adherence to malaria diagnosis guidelines as reported by patients

Variable	Frequency	Per cent
Healthcare provider asked about symptoms of the illness		
No	10	5.1
Yes	187	94.9
Total	197	100.0
Healthcare provider asked if the patient has fever/history of fever		
No	39	19.8
Yes	158	80.2
Total	197	100.0
Healthcare provider asked about any signs of severe illness (headache, convulsions, unable to eat or drink, vomiting)		
No	102	51.8
Yes	95	48.2
Total	197	100.0
Healthcare provider asked for patient’s age		
No	6	3.0
Yes	191	97.0
Total	197	100.0
Healthcare provider weighed patient using a weighing scale		
No	140	71.1
Yes	57	28.9
Total	197	100.0
Healthcare provider took body temperature with a thermometer		
No	153	77.7
Yes	44	22.3
Total	197	100.0
Healthcare provider took both medical history and physical examinations appropriately		
No	181	91.9
Yes	16	8.1
Total	197	100.0
Provider recommended/offered a blood test (RDT and microscopy) for malaria (even if patient refused test)		
No	68	34.5
Yes	129	65.5
Total	197	100.0
Patient, among those offered a test, actually took the test (n = 129)		
Yes	129	100.0
No	0	0.0
Total	129	100.0
Type of test done (n = 129)		
RDT	124	96.1
Microscopy	5	3.9
Total	129	100.0
Proportion of providers who fully adhered to malaria diagnosis guidelines^a^		
No	185	93.9
Yes	12	6.1
Total	197	100.0

Source: primary data

^a^ Full adherence refers to patients whose providers adhered to all medical history taking questions, physical examinations questions and malaria laboratory testing

In terms of laboratory diagnosis, 129 (65.5%) of the patients were recommended for malaria testing and all (100.0%) of them went ahead to have the test done. Among the patients who actually took the test, RDT was the most common type of malaria testing done (96.1%) and only 3.9% had a microscopy test. Additionally, only about half (52.4%) of the 124 patients who had RDT done had a positive test result and 80.0% of those who took microscopy had a positive blood smear test for malaria.

The findings indicate that the overall level of adherence to malaria diagnosis (medical history, physical examination, and laboratory testing) was only 6.1% of the 197 patients.

### Level of adherence to malaria drug (ACT) prescriptions guidelines

In total, 127 (64.5%) of the 197 patients who participated in this study received ACT drugs or ACT drug prescription as shown in Table 3. 63.0% of patients who received ACT drugs or prescription had received it after a malaria test. The results show that 24 (18.6%) of the patients who had negative malaria test were still given ACT drug prescriptions, while 13 (10.1%) who had positive test results were not given ACT prescriptions by the providers.

**Table 3:** Level of adherence to malarial drug (ACT) prescriptions guidelines, reported by patients

Variable	Frequency	Per cent
Patient received ACT drug or ACT drug prescription		
No	70	35.5
Yes	127	64.5
Total	197	100.0
Patient, among those who received ACT drug/prescription, received ACT drug/prescription after a malaria test (n = 127)		
No	47	37.0
Yes	80	63.0
Total	127	100.0
Test (RDT and microscopy) results and ACT drug prescriptions		
Negative (did not receive ACT prescription)	36	27.9
Negative (received ACT drug prescription)	24	18.6
Positive (did not receive ACT drug prescription)	13	10.1
Positive (received ACT drug prescription)	56	43.4
Total	129	100.0
Overall proportion of patients whose providers adhered to national IMM guidelines (among all n = 197)^a^		
No	191	97.0
Yes	6	3.1
Total	197	100.0

Source: primary data

^a^ Overall proportion of adherence to national malaria management was computed and means that the providers adhered to both malaria diagnosis and prescriptions guidelines. Prescription adherence meant that a provider prescribed anti-malarial after testing for malaria and only to those who tested positive. Prescriptions of ACT to patients without testing for malaria and to those with negative test results constituted non-adherence to prescription and were factored in computing for overall level of adherence to guidelines

Overall, the level of adherence to the national malaria management guidelines (clinical, laboratory diagnosis, prescription) among the patients was very low at only 3.1% of the patients being managed in accordance with IMM guidelines.

### Provider-related factors and adherence to national malaria management guidelines

Patients reported 26 different providers among the 11 health facilities. As shown in Table 4, there were significant differences between the levels of adherence for providers who had training on national malaria treatment guidelines in the past 3 years and those who did not have the training (X^2^ = 7.564, p = 0.014) as well as among the professional cadres (X^2^ = 27.468, p < 0.001). There was no significant difference in the level of adherence to the guidelines as far as the other provider-related factors were concerned. Furthermore, there was no significant difference in the level of adherence between those who expressed positive perceptions and those with negative perceptions about the key components of IMM guidelines (p > 0.05).

**Table 4:** Provider-related factors and adherence to national malaria management guidelines

Provider demographics (n = 26)	Reported adherence to IMM guidelines		Chi square test (X^2^) statistic (df)/FET	p-value
Gender	No	Yes		
Female	16 (88.9)	2 (11.1)	0.821 (1)	0.563
Male	6 (27.3)	2 (50.0)		
Age of providers				
19–24 years	4 (18.2)	0 (0.0)		
25–30 years	12 (54.5)	3 (75.0)	9.996	0.694
31–36 years	3 (13.6)	0 (0.0)		
37 years and above	3 (13.6)	1 (25.0)		
Years worked in current position				
0–5	20	3		
6–10	2	0	7.239 (5)	0.203
Over 10	0	1		
Training the last 36 months on the national treatment guidelines for malaria?**				
No	16 (72.7)	0 (0.0)	7.564	0.014
Yes	6 (27.3)	4 (100)		
Highest level of education attained				
Primary education	7 (31.8)	2 (50.0)		
Secondary education	6 (27.3)	0 (0.0)	3.381	0.336
Some university	1 (4.5)	1 (25.0)		
University/college	8 (36.4)	1 (25.0)		
Healthcare provider cadre**				
Health Assistant/Nursing Assistant/Nursing Aide	8 (36.4)	1 (25.0)		
Community Medicine Distributor/Village Health Team	1 (4.5)	0 (0.0)	27.468 (7)	< 0.001
Clinical Officer	1 (4.5)	1 (25.0)		
Nurse/Nursing Officer	7 (31.8)	1 (25.0)		
Midwife	3 (13.6)	1 (25.0)		
Laboratory technician/Laboratory assistant	2 (9.1)	0 (0.0)		
Provider knowledge about first-line anti-malarials for uncomplicated malaria				
Correctly identified first-line medications	17 (77.3)	4 (100.0)	1.600 (2)	0.449
Incorrectly identified first-line line medications	4 (18.2)	0 (0.0)		
Admitted did not know	1 (5.5)	0 (0.0)		
Provider perceptions towards IMM guidelines				
If a patient comes with mild fever, he/she must be tested before any treatment				
Agree	20 (90.9)	4 (100.0)	0.688 (1)	0.407
Disagree	2 (9.1)	0 (0.0)		
Sometimes RDTs do not provide correct results				
Agree	16 (72.7)	3 (75.0)		
Disagree	5 (22.7)	0 (0)	2.368 (2)	0.306
Neither agree nor disagree	1 (4.6)	1 (25.0)		
I do not trust results of RDT				
Agree	17 (77.3)	3 (75.0)		
Disagree	4 (18.2)	1 (25.0)	1.143 (2)	0.565
Neither agree nor disagree	1 (4.5)	0 (0.0)		
There are times when I treat patients with an anti-malarial even when they test negative for malaria as long as they have symptoms for malaria				
Agree	19 (86.4)	4 (100.0)	0.811 (1)	0.368
Disagree	3 (13.6)	0 (0.0)		
It is ‘okay’ to treat a patient with fever without testing				
Agree	7 (31.8)	1 (25.0)	0.015 (1)	0.902
Disagree	15 (68.2)	3 (75.0)		

Source: primary data

** Statistically significant variable

### Facility-related factors and adherence to national malaria management guidelines

The results in Table 5 show that none of the providers from public health facilities had their patients report adherence to the national treatment guidelines for malaria while 75% of all the providers whose patients reported adherence were in private not-for-profit facilities. There was a statistically significant association between facility ownership and the level of adherence to malaria treatment guidelines (X^2^ = 13.517, p = 0.001).

**Table 5:** Facility-related factors and adherence to national malaria management guidelines

Variable	Reported adherence to IMM guidelines		Chi square test (FET)	p-value
Level of facility (n = 26)	No	Yes		
Level II	11 (50.0)	1 (25.0)		
Level III	11 (50.0)	3 (75.0)	0.851	0.598
Total	22 (100.0)	4 (100.0)		
Facility ownership**				
Private	7 (31.8)	1 (25.0)		
Public	14 (63.6)	0 (0.0)	13.517	0.001
Private not-for-profit (PNFP)	1 (4.5)	3 (75.0)		
Total	22 (100.0)	4 (100.0)		

Source: primary data

** Statistically significant variable

Table 5 also shows that the level of facility was not a statistically significant factor in reported adherence to IMM guidelines. Level II facilities are the “lowest level of formal healthcare delivery” and are “mostly staffed by nurse aides and qualified nurses,” whereas Level III facilities are mid-level primary care facilities that provide for “basic laboratory services, maternity care and inpatient care (often for onward referral)” and are “usually staffed by nurse aides, qualified nurses and clinical officers (physician assistants)” [10]. These types of health facilities focus on preventing and treating infectious illnesses.

### Multivariate analysis of factors associated with adherence to national malaria management guidelines

Multivariate analysis was conducted for highly significant variables (professional cadre, whether the provider receiving training on IMM guidelines in the past 3 years, ownership of facility). From Table 6, only training in the last 3 years (OR = 2.858, 95% CI 1.754–4.659, p < 0.001) was independently associated with adherence to the national malaria management guidelines at the multivariate level. Health workers who received training on the national malaria management guidelines in the past 3 years were about three times more likely to adhere to the guidelines compared to those who did not. There was no significant difference in the levels of adherence to the guidelines by professional qualification and ownership of the facility (p > 0.05).

**Table 6:** Multivariate analysis of factors associated with adherence to national malaria management guidelines

Variable	Odds ratio (OR)	95% CI for OR		p-value
Professional cadres (n = 26)		Lower	Upper	
Clinical officer	1.000			
Enrolled nurse/nursing officer	1.891	0.030	118.772	0.763
Midwives	0.946	0.015	59.386	0.979
Laboratory technician/assistant	1.788	0.004	885.281	0.854
Health Assistant/Nursing Assistant/Nursing Aid	1.891	0.030	118.772	0.763
Received training on IMM guidelines in the past 3 years** (n = 26)				
Yes	2.858	1.754	4.659	< 0.001
No	1.000			
Ownership of the facilities (n = 26)				
Private	0.006	0.002	0.094	0.086
Government	0.026	0.003	0.193	0.251
Private not for profit	1.000			

Source: primary data

** Statistically significant variable

## Discussion

This is one of the first studies to have assessed the level of adherence to national malaria management guidelines by healthcare workers in Kaliro and Buyende districts, a lakeshore area with high malaria burden in Uganda. Given its ecological nature, the area provides favorable mosquito breeding sites, which may partly explain the high malaria burden. At the same time, this natural quality emphasizes the need for more stringent measures to combat the high burden. Adherence to IMM guidelines by health workers is one of the vital measures that ought to front this fight.

### Level of adherence to malaria diagnosis guidelines

The proportion of patients/caregivers who reported overall adherence to both appropriate medical history taking (being asked about age, when fever started, symptoms including those of severe illness) and physical examination (having weight and temperature taken) was 8.1%. This finding is much lower than the number reported among children in Bolgatanga Hospital, Ghana which reported high adherence to physical examinations, with 95% having their body temperature and 94% having their respiratory and pulse rate taken as recommended in the Ghana national malaria management guidelines [11]. One possible explanation for this stark difference could be due to Uganda’s high client load, which may overwhelm providers and deter them from following the appropriate steps in diagnosis. Other potential reasons include inadequate medical equipment, such as weighing scales and thermometers.

The 65.5% of patients in this study who were recommended to do a malaria test is lower than that found in a study in Ghana, in which 76% of the patients were requested to have laboratory tests done prior to treatment for malaria [11]. This difference could be due to the differences in provider knowledge and perceptions related to malaria diagnosis. For instance, health providers in Uganda believe in clinical diagnosis of malaria rather than testing for the disease [12], so many may not prioritize testing and instead opt to use clinical signs. This preference may be attributed to previous Ministry of Health policies, which condoned diagnosis for malaria based on clinical signs [12]. This observation may also imply that it may take some time for health workers to fully adopt the test-and-treat guidelines, even if current policies shift.

The overall level of malaria diagnosis (laboratory testing and clinical diagnosis) was only 6.1%, which is not in line with the current National Malaria Control Policy in Uganda. This policy recommends microscopy or RDT for confirmation of all suspected malaria cases before prescribing any anti-malarial [3]. It is a deplorable finding, given that poor adherence to the diagnostic guidelines has a direct implication on the treatment given and subsequently the outcome of such treatments [12].

### Level of adherence to malaria prescription guidelines

The finding about adherence to prescription guidelines indicates that close to 65% of the patients who participated in this study received ACT drug or ACT drug prescription even though only 54% of those tested were positive. This is not surprising, given that in the provider interviews, most providers agreed that they would go ahead and treat a patient with anti-malaria drug even when the test results were negative. This finding is consistent with the earlier findings of a similar study among public health workers in the Kamuli district, which found that health providers prescribed anti-malarial drugs for almost all patients who presented having fever without doing confirmatory laboratory tests [13]. This action contradicts the national malaria management guidelines that require testing of all suspected cases of malaria prior to treatment with anti-malarial drugs [3]. Furthermore, this finding also reflects provider practices in relation to malaria management across sub-Saharan Africa. Another study in Ghana found 98% non-adherence to the national prescription guidelines with regard to prescription of first-line anti-malarial drugs for management of severe malaria [11].

Of major concern, 40% of patients with negative malaria test results in this study received ACT drug or other anti-malarial drug prescription. This result implies that health workers follow the clinical diagnosis guidelines and use fever as the major symptom of malaria [11]. A similar finding was reported in a retrospective audit of patient records in two public hospitals in Nigeria and showed that 58% of patients who tested negative received anti-malarial drugs [14]. These findings portray similarity in health provider practices between African countries. Study findings were also consistent with studies in other developing countries by indicating that inappropriate prescription and use of ineffective anti-malarial drugs persists in both public and private health facilities [15] and substantial inappropriate treatment practices, including treating people with negative malaria test results [16]. Policy makers ought to focus on dispelling the belief that fever is an indicator of malaria infection. This can be done through refresher training on the new policy guidelines. Similarly, 19% of patients with a positive test result for malaria were not given ACT drug or any other prescription for anti-malaria drugs, which also defies the current treatment guidelines for malaria that require recommending an ACT for every uncomplicated malaria case. The study was not able to establish why patients with positive results were not prescribed or given an ACT.

The observed 3.1% overall level of adherence to IMM guidelines is extremely low, especially for lakeshore areas such as Buyende and Kaliro. This finding may translate into a larger public health concern since non-adherence can lead to drug resistance, resource waste, adverse health outcomes including death, and lower healthcare quality by health facilities [17]. The low level of adherence may be due to negative provider perceptions; an astonishing 88% of health workers interviewed in this study agreed that they treat patients with anti-malarial even though they have negative test results.

### Factors influencing adherence to the IMM guidelines

Training on how to use national malaria treatment guidelines, the professional cadre of the healthcare providers, and facility ownership were the only factors that were significantly associated with adherence to malaria management guidelines. At the same time, IMM training in the past 3 years was the only factor that independently influenced adherence. This result implies that training healthcare providers on national malaria management guidelines should improve their levels of adherence to the guidelines. However, a study among public health facilities in the Kamuli district in Eastern Uganda contradicts this finding. It found no statistically significant association between attending malaria-specific continuing medical education (CME) sessions and increased adherence to the national malaria treatment guidelines [13]. This contradiction may be due to the difference in the definition of training between this study and the study in Kamuli. Another explanation could be because the Kamuli study was solely among public health facilities where training may be routine and identical for all health workers, whereas the present study was also conducted in private health facilities where training on IMM may not be routine.

### Study limitations

One limitation is that adherence to IMM guidelines was patient-reported rather than observed. This aspect could have caused bias in the information provided by patients. However, the researchers believe that there were few instances of bias since patient respondents would lack any negative motives towards health workers or may not be sure of what healthcare workers should or should not be doing. It is believed that being patient-reported did not affect the results.

Another limitation is that the number of health workers was very low, which could have impacted the measurement of the factors that may influence adherence to the IMM guidelines. Interviewing more health workers would have required covering more area, since the number of health workers in Uganda in generally low, and consequently more funding and resources. On the other hand, the number considered in this study may be representative of the healthcare workers in the lakeshore areas of Kaliro and Buyende districts.

In addition, the cross-sectional nature of this study poses a limitation in that inference between IMM adherence and associated factors is difficult to make. A longitudinal study should be considered in future to make further conclusions on this subject.

## Conclusions

The overall level of adherence to the national malaria management guidelines in Kaliro and Buyende districts was very low, with only 3.1% of the patients reported being treated in line with national IMM guidelines. Training in the last 3 years was the only factor that independently influenced adherence to the national malaria management guidelines among the providers. Policy makers ought to focus on dispelling the belief that fever is an indicator of malaria infection. This can be done through refresher training on new policy guidelines.
